# Endothelial Toxicity of High Glucose and its by-Products in Diabetic Kidney Disease

**DOI:** 10.3390/toxins11100578

**Published:** 2019-10-05

**Authors:** Laetitia Dou, Noémie Jourde-Chiche

**Affiliations:** 1 Aix Marseille University, INSERM, INRA, C2VN, Faculté de Pharmacie, 27 Bd Jean Moulin, 13005 Marseille, France; noemie.jourde@ap-hm.fr; 2 Centre de Néphrologie et Transplantation Rénale, AP-HM, Hôpital de la Conception, 147 Bd Baille, 13005 Marseille, France

**Keywords:** AGEs, diabetic kidney disease, endothelial dysfunction, glucose, polyols

## Abstract

Alterations of renal endothelial cells play a crucial role in the initiation and progression of diabetic kidney disease. High glucose per se, as well as glucose by-products, induce endothelial dysfunction in both large vessels and the microvasculature. Toxic glucose by-products include advanced glycation end products (AGEs), a group of modified proteins and/or lipids that become glycated after exposure to sugars, and glucose metabolites produced via the polyol pathway. These glucose-related endothelio-toxins notably induce an alteration of the glomerular filtration barrier by increasing the permeability of glomerular endothelial cells, altering endothelial glycocalyx, and finally, inducing endothelial cell apoptosis. The glomerular endothelial dysfunction results in albuminuria. In addition, high glucose and by-products impair the endothelial repair capacities by reducing the number and function of endothelial progenitor cells. In this review, we summarize the mechanisms of renal endothelial toxicity of high glucose/glucose by-products, which encompass changes in synthesis of growth factors like TGF-β and VEGF, induction of oxidative stress and inflammation, and reduction of NO bioavailability. We finally present potential therapies to reduce endothelial dysfunction in diabetic kidney disease.

## 1. Introduction

Diabetic kidney disease (DKD) is a complication occurring in patients with diabetes, characterized by the chronic loss of kidney function. DKD is the leading cause of chronic kidney disease (CKD) in developed countries [1,2]. It is characterized clinically, in patients with both type 1 and type 2 diabetes, by kidney enlargement and hyperfiltration, followed by microalbuminuria, macroalbuminuria, and progressive CKD associated with hypertension [3]. Alterations of renal endothelial cells play a major role in the initiation and progression of DKD [4]. 

Different types of endothelial cells are present in the kidney, including glomerular endothelial cells (GECs), microvascular endothelial cells within peritubular capillaries, and endothelial cells within larger venous and arterial blood vessels [5]. Endothelial cells from the renal microcirculation actively communicate with other cells, notably pericytes, which are localized in the tubulointerstitial space surrounding peritubular capillaries, in the afferent and efferent arterioles, and within the glomerulus as mesangial cells [4]. In glomeruli, endothelial cells are also in close interaction with specialized epithelial cells, namely the podocytes, which contribute to the glomerular filtration barrier.

The glomerular filtration barrier, which allows the selective filtration of water, glucose, electrolytes, and small molecules, and restrains the passage of albumin and blood cells, is comprised of three layers: the fenestrated glomerular endothelium, the glomerular basement membrane, and the interdigitated foot processes of podocytes separated by the podocyte filtration slits [4]. GECs are thus involved in glomerular filtration whereas endothelial cells of peritubular capillaries are involved in tubular secretion and reabsorption. Both GECs and peritubular endothelial cells are fenestrated, although ultrastructural differences exist between them [6]. The fenestration of GECs and peritubular endothelial cells is critical for the permselectivity of the glomerular filtration barrier and for the efficient passage of high volumes of fluids and the formation of urine. GECs are lined by a particularly thick filamentous glycocalyx that is enriched in negatively charged proteoglycans (mostly heparan and chondroitin sulfate) that form a network with glycosaminoglycans (mostly hyaluronic acid). The glycocalyx contributes to the regulation of vascular permeability and fluidic balance and repels blood cells away from the vascular wall [7,8].

Furthermore, the glomerular filtration is controlled by hemodynamic changes within the renal afferent and efferent arterioles, whose vascular tone is essentially regulated by vasodilators like prostaglandins and nitric oxide (NO), and by the renin-angiotensin system (RAS). 

**Figure 1:**
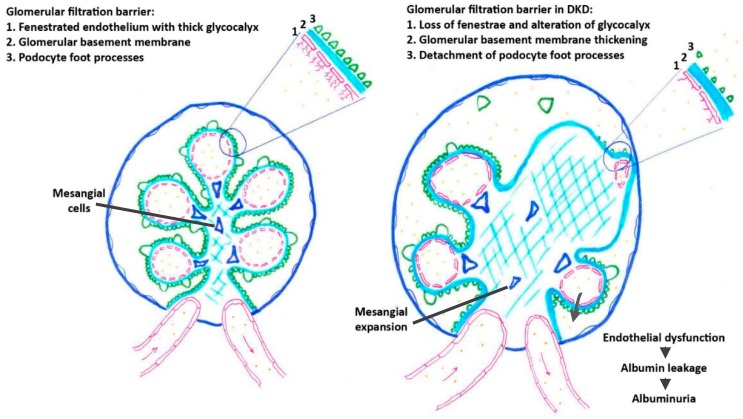
Pathological changes affecting glomerular filtration barrier in DKD.

Pathologically, DKD is characterized by mesangial deposition of extra-cellular matrix, glomerular basement membrane thickening, and glomerulosclerosis [3]. DKD is also characterized by tubular atrophy and interstitial fibrosis with peritubular capillary rarefaction, with the involvement of endothelial-mesenchymal transition (EndMT) [9].Major changes are observed in fenestration and glycocalyx of GECs, along with endothelial cell loss and capillary rarefaction in both the glomeruli and tubulointerstitium [9]. A progressive reduction in the surface of fenestrated endothelium is observed in diabetic patients as DKD progresses. In patients with type 2 diabetes, the reduction in glomerular endothelial fenestrae correlates with albuminuria and loss of glomerular filtration rate (GFR) [10]. Microalbuminuria is a hallmark of renal as well as systemic endothelial dysfunction [11]. In addition to the modification of GEC fenestrae, an alteration in endothelial glycocalyx occurs in DKD (Figure 1). Endothelial dysfunction is associated successively with glomerular enlargement with glomerular basement membrane thickening, mesangial expansion, podocyte detachment (Figure 1), the development of glomerulosclerosis [12], tubular atrophy, and interstitial fibrosis [13].

## 2. Effect of High Glucose and by-Products on Renal Endothelial Cells Leading to DKD

### 2.1. Toxicity of High Glucose and Glucose by-Products 

In diabetes, high glucose leads to the formation of glucose by-products like AGEs and metabolites produced via the polyol pathway. 

#### 2.1.1. Glucose

The toxic effect of high glucose per se on endothelial cells is well-established. High glucose induces endothelial dysfunction in both large vessels and the microvasculature. The autoregulation of glucose uptake is different in macro- and microvascular endothelial cells as well as endothelial cells from different organs [14,15]. For example, microvascular retinal endothelial cells do not decrease glucose uptake when exposed to high extracellular glucose concentrations, whereas brain- and heart-derived endothelial cells do [14]. One can suppose that a particular regulation of glucose uptake exists in GECs, like in retina endothelial cells, which can explain their increased susceptibility to injury when exposed to high glucose for prolonged periods.

#### 2.1.2. The Polyol Pathway

**Figure 2:**
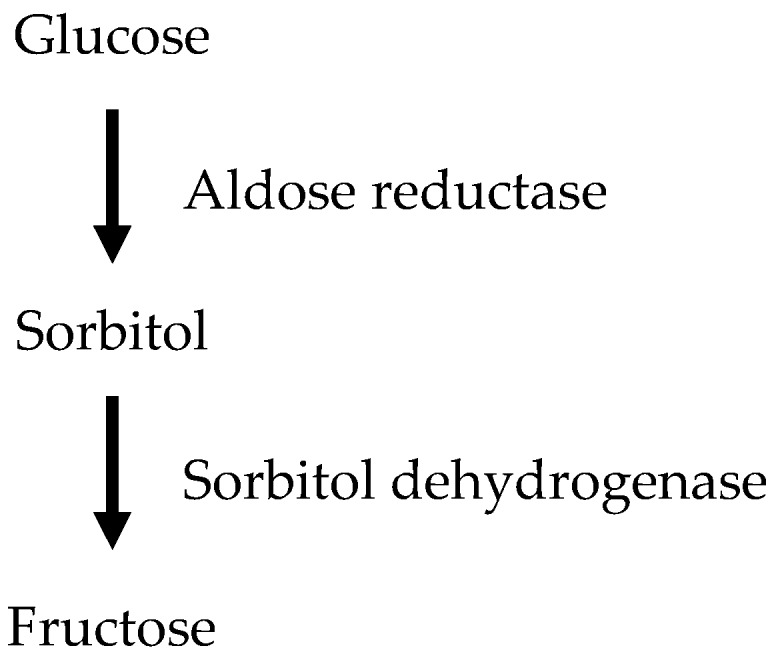
The polyol pathway. The polyol pathway is a two-step enzymatic pathway. In the first strep, glucose is reduced to sorbitol by aldose reductase. In the second step, sorbitol is converted to fructose by sorbitol dehydrogenase. Endothelial toxicity arises from sorbitol/fructose accumulation within endothelial cells.

Hyperglycemia activates the polyol pathway, a two-step metabolic pathway in which glucose is reduced to sorbitol by aldose reductase, which is then converted to fructose by sorbitol dehydrogenase (Figure 2). This increase in glucose metabolism via the polyol pathway results in the accumulation of intracellular sorbitol, which may contribute to the development of microvascular dysfunction in diabetes. The pathogenic role of the polyol pathway was shown by inhibition of aldose reductase that attenuates proteinuria, decreased glomerular basement membrane thickening in diabetic rats, and reduced glomerular hyperfiltration in humans [4].

#### 2.1.3. Advanced Glycation End Products (AGEs)

**Figure 3:**
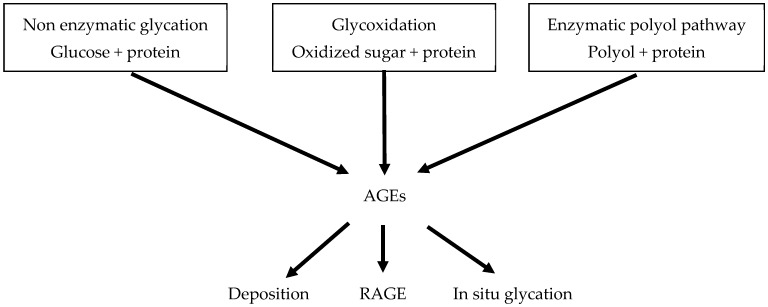
The major biomechanisms leading to the formation of advanced glycation end products (AGEs).

Chronic hyperglycaemia promotes the formation of AGEs, a group of modified proteins and/or lipids that become glycated after exposure to sugars. The formation of AGEs can occur through three major biochemical mechanisms: non-enzymatic glycation, in which glucose binds to a protein in a high-glucose-concentration environment; the enzymatic polyol pathway wherein sorbitol/fructose from aldolase reductase- or sorbitol dehydrogenase-converted glucose bind to a protein; and glycoxidation, whereby the oxidation of glucose leads to the formation of oxidized sugars glyoxal and methylglyoxal that can react with different proteins [16] (Figure 3). AGE toxicity is linked to three mechanisms: deposition, in situ glycation, and AGE–receptor interaction [16] (Figure 3). At least five types of AGE receptors have been identified. The most widely studied is RAGE [16], which is upregulated in endothelial cells by hyperglycemia [4].

### 2.2. Mechanisms Inducing Endothelial Dysfunction Leading to DKD

#### 2.2.1. Effect of High Glucose and by-Products on Glomerular Filtration Barrier

High glucose and its by-products may impair the glomerular filtration barrier in three major ways: (1) increased permeability of GECs, (2) alteration of glycocalyx, (3) induction of GEC apoptosis. 

##### Endothelial Permeability

High glucose increases the permeability of GECs [17], particularly via protein kinase C (PKC) activation [11]. PKC is predominantly activated by diacylglycerol increased by hyperglycemic conditions [11]. PKC inhibition reduces hyperglycemia-induced hyperpermeability in different endothelial cells, notably glomerular [11]. In cultured endothelial cells, PKCα, and to a lesser extent PKCβ, appear to be the isoforms involved in hyperglycemia-induced hyperpermeability [11]. In diabetic rats, inhibition of PKCβ reduces albumin excretion, and both glomerular and tubulointerstitial injury [18]. PKC activation induced by high glucose up-regulates the Cyclooxygenase- 2 (COX-2) in endothelial cells [19]. COX-2 up-regulation is associated with an imbalance in eicosanoids profile, with an increase in vasoconstricting thromboxane B2 and a decrease in vasodilatory 6-keto-prostaglandin Fα [19]. These changes in prostanoids production, leading to renal hemodynamic changes, may participate in glomerular hyperfiltration observed in early DKD. Activation of the RAS could be involved in GEC alterations leading to hyperpermeability [20]. Indeed, GECs exposed to high glucose display significant RAS activation, associated with increased GEC fenestration, and higher permeability to albumin. All these changes were abrogated by angiotensin-receptor blockers [20]. 

In DKD, GEC injury leading to endothelial surface layer degradation may results from pathologic crosstalk between activated podocytes and GECs, as shown in experimental focal segmental glomerulosclerosis [21]. 

##### Glycocalyx Alteration

The biosynthesis of glycosaminoglycans (especially heparan sulfate) is markedly reduced in GECs exposed to high glucose [22], with an increased passage of albumin through GECs monolayers [22]. An inverse relationship between proteinuria and heparan sulphate-associated anionic sites in the glomerular basement membrane is observed in rats with streptozotocin-induced DKD [23]. The synthesis of heparanase, the enzyme that degrades heparan sulphate, both by GECs and by podocytes, is increased in glomeruli of patients with DKD [24]. This synthesis results from high glucose-induced production of ROS and angiotensin II in GECs [25], as well as from the nuclear factor-kappa B (NF-κB) pathway activation by the receptor of AGEs in podocytes [26]. Mice lacking heparanase are protected from DKD after streptozotocin-induced diabetes [27]. Treatment of diabetic mice with an heparanase inhibitor reduces albuminuria and kidney injury [27]. However, no therapy targeting the glycocalyx has proven beneficial in patients with DKD yet [28,29]. 

##### Endothelial Apoptosis 

High glucose increases the apoptosis of GECs [17], via PKC activation that leads to pro-apoptotic protein up-regulation [30]. RAS activation is also probably involved. Indeed, in rat GECs, high glucose modifies the expression and localization of angiotensin II receptors, and increases angiotensin II generation [31], which is associated with increased endothelial cell apoptosis [9].

The concomitant alterations of genes involved in the regulation of apoptosis and oxidative stress in GECs from diabetic mice [32] support a role of increased oxidative stress in glomerular EC apoptosis. The increase in intracellular glucose leading to increased generation of reactive oxygen species (ROS) could trigger c-Jun NH2 terminal kinase (JNK) activation that finally mediates caspase 3-induced apoptosis of endothelial cells [33]. 

#### 2.2.2. Changes in VEGF Pathway

Podocytes play a crucial role in the preservation of GEC structure and function, through the synthesis of angiogenic factors like Vascular Endothelial Growth Factor (VEGF) [6,9]. The canonical signaling of VEGF within the renal glomerulus involves the secretion of VEGF by podocytes, which then binds to VEGFR2 on the surface of GECs, after passing counter flow through the filtration barrier [34]. 

In DKD, VEGF synthesis is dysregulated. Both up- and downregulations of VEGF have been reported in the kidney from patients and rodents with diabetes [9,34,35]. While an initial upregulation of VEGF pathway may occur in early stages of DKD in response to endothelial stress, subsequent reduced VEGF expression could result from diminished podocyte number because of DKD progression [34]. In patients with diabetes, the binding of VEGF to its endothelial receptors depends on the level of glomerular injury; it is increased in mildly injured glomeruli, and decreased in more severely injured glomeruli [35]. Hyperglycemia and AGEs affect the renal VEGF pathway by decreasing the expression of VEGFR2 on GECs [34]. This occurs via the activation of the TGF-β alternative signaling pathway [36], and leads to a reduced responsiveness of GECs to VEGF [34]. In diabetic mice, pharmacological inhibition of VEGF signaling promotes endothelial injury and accelerates the progression of glomerular lesion [4]. The initial upregulation of VEGF may participate in increased glomerular permeability in early stages of DKD, whereas subsequent downregulation may hamper effective capillary repair in advanced stages [4,35]. 

#### 2.2.3. Synthesis of TGF-β

Many researches about the pathogenesis of DKD have focused on transforming growth factor- β (TGF-β), an important pro-fibrotic and anti-inflammatory factor [37]. In diabetic animal models and in patients with DKD, hyperglycemia and AGEs stimulate TGF-β secretion [4,38]. Activation of the TGF-β canonical pathway via Smad-2/3 signaling leads to extracellular matrix deposition and increased glomerular barrier membrane thickness [38]. In renal endothelial cells, TGF-β1 mediates renal fibrosis by inducing modifications characteristic of endothelial–mesenchymal transition (EndMT) [39,40], a phenomenon whereby endothelial cells begin to express the characteristics of mesenchymal cells, with the acquisition of mesenchymal markers like α-smooth muscle actin (αSMA) [9] and loss of endothelial markers like VE-cadherin and CD31 [9,40]. TGF-β was a promising therapeutic target in animal models of DKD [41]. However, results of TGF-β inhibition were disappointing in humans [42].

#### 2.2.4. Oxidative Stress and Reduced NO Bioavailability

Diabetic patients display an impaired NO-mediated vasodilation [4] and an increased oxidative stress, resulting from increased ROS production and from reduction of endogenous antioxidant systems [43].

Reduced NO bioavailability has a pivotal role in renal endothelial injury in DKD [44]. NO produced by GECs is a protective factor for podocytes, and the deficiency of endothelial nitric oxide synthase (eNOS) increases podocyte injury and accelerates DKD in mice [5]. High glucose reduces NO production by human endothelial cells [45], and AGEs reduce eNOS expression and inactivate NO [16]. In patients with DKD, the elevation of ADMA (Asymmetric Dimethylarginine) [46], the endogenous inhibitor of eNOS, may also participate in eNOS inhibition [4].

Under physiological conditions, eNOS functions as a homodimer, and homodimer uncoupling leads to superoxide anion (O_2_^−^) formation instead of NO production. The production of O_2_^−^ instead of NO further reduces NO bioactivity, and promotes the formation of peroxynitrite [4]. The active dimeric form of eNOS is phosphorylated at Serine 1179/1177. In cultured mouse GECs exposed to high glucose, and in glomeruli of diabetic mice, there is a significant decrease in the dimerized form of eNOS [17]. A decrease in eNOS phosphorylation was demonstrated in diabetic mouse glomeruli [17], in moderately hyperglycemic diabetic rats [47], as well as in high glucose-treated GECs [17]. Furthermore, optimal concentrations of the eNOS substrate, L-arginine, and the co-factor tetrahydrobiopterin (BH_4_) are crucial to maintain eNOS dimerization leading to NO production [4]. A profound depletion of BH_4_ level has been observed in microvascular endothelial cells isolated from diabetic rats [4]. Treatment of GECs with L-arginine or the BH4 cofactor, sepiapterin, partially corrected the impairment of eNOS dimerization, phosphorylation, and activity induced by high glucose [17]. Yet, oral supplementation with L-arginine failed to prevent or reduce renal injury in diabetic mice [48].

Increased oxidative stress plays an important role in renal endothelial damage. In GECs, increase in ROS production due to high glucose relies on PKC-dependent activation of NAD(P)H oxidase, and on other enzymatic sources like xanthine oxidase and eNOS [45,49]. High glucose also increases O_2_^−^ production by the mitochondria of endothelial cells [50]. The production of O_2_^−^ further reduces NO bioavailability by promoting the formation of peroxynitrite ONOO^−^ [4]. ROS-stimulated formation of peroxynitrite was shown in patients with type 2 diabetes and in diabetic mice. Peroxynitrite can also nitrate tyrosine residues of proteins, leading to 3-nitrotyrosine formation. An increased arterial O_2_^−^ production associated with the formation of 3-nitrotyrosine was found in high-sorbitol-exposed rat arterioles [51]. ROS promote the formation of AGEs, which, in turn, bind to their receptor RAGE, and further increase ROS synthesis in endothelial cells, through activation of the NADPH-oxidase pathway [16,52]. 

In rats, increased oxidative stress induces the deterioration of the glomerular endothelial surface layer [53]. The specific involvement of mitochondrial ROS in endothelial damage has been nicely demonstrated in early diabetic mice [54]. DKD-susceptible mice and DKD patients display mitochondrial DNA lesions in glomerular endothelial cells, associated with increased glomerular endothelin-1 receptor type A (Ednra) expression [54]. In mice, diabetes-induced endothelial injury, albuminuria, podocyte loss, and glomerulosclerosis are ameliorated by scavenging of mitochondrial ROS or selective Ednra blockade, which prevent endothelial mitochondrial oxidative stress [54]. Increased ROS production, in association with decreased NO bioavailability, upregulates COX-2 gene transcription [45,55], and modifies the production of prostanoids [45,51,55] that control vascular reactivity of the renal glomerular arterioles. High glucose-induced ROS generation play a central role in extracellular matrix synthesis via up-regulation of pro-fibrotic factors like TGFβ1 and angiotensin II, leading to renal fibrosis [56]. 

#### 2.2.5. Proinflammatory Effects

High glucose and AGEs may promote inflammation by upregulating the expression of endothelial molecules involved in leukocyte adhesion like VCAM-1, E-selectin, and ICAM-1 [4,57]. Leukocytes from diabetic animals exhibit elevated levels of counter-receptors of these endothelial adhesion molecules [58]. Consequently, the adhesion of diabetic leukocytes on microvascular endothelial monolayers is enhanced [58]. Endothelial inflammation mediated by high glucose could be related to stimulation of the proinflammatory transcription factor NF-κB by PKC and ROS [50]. The increased levels of inflammatory cytokines present in diabetic patients could also participate in induction of endothelial inflammatory molecules [4]. 

#### 2.2.6. Defect of endothelial progenitor cells

The microvascular rarefaction and endothelial loss characteristic of diabetes are associated with defective endothelial repair that involves endothelial progenitor cells (EPC). In the literature, the term "EPC" has encompassed different types of cells whose common point is to promote vascular repair (see [59] for review). These cells include angiogenic cells of myeloid origin, endothelial colony forming cells, as well as "true" EPCs [59,60]. Whatever the type of EPC considered, studies have reported reduced numbers in diabetic patients [60]. EPCs from diabetic patients exhibit decreased repair capacities, with an impaired ability to proliferate and a defect in incorporation in vascular structures [9,61,62]. Impaired EPC migration in diabetes could be linked to a downregulation of the CXCR4/Pi3K/Akt/eNOS signaling pathway [63]. Diabetic EPCs display reduced eNOS expression and decreased NO bioavailability, as well as increased NADPH oxidase activity and superoxide levels [64]. NADPH oxidase inhibition in diabetic cells restored migratory function in vitro and enhanced their homing to ischemic retinal vasculature in vivo [64]. 

The high glucose environment associated with diabetes impairs all processes involved in maintaining adequate numbers and function of EPCs. High glucose significantly enhances the senescence of EPC from healthy subjects [63], and reduces their angiogenic properties [63]. The culture, in normal glucose for seven days, failed to restore the angiogenic properties of progenitor cells from diabetic rats and their secretion of proangiogenic chemokines, supporting the hypothesis of a metabolic memory [65]. 

The deleterious impact of AGEs on EPC is also clearly established. In healthy subjects, serum levels of AGEs are independently correlated with reduced number of EPCs [66]. In vitro, AGEs promote EPC apoptosis [67], by downregulating the anti-apoptotic factor Bcl-2, and increasing Caspase-3 expression [68]. Incubation of EPCs with AGEs impairs EPC migration, adhesion, and tube formation, in a concentration-dependent manner [67]. AGE-induced impairment of EPC function is mediated by RAGE, down-regulation of protein kinase Akt [67], and by decreased production of the stromal cell-derived factor 1 (SDF-1) chemokine [69]. AGEs can also progressively modify the vascular basement membrane, thus contributing to impairment of EPC reparative function. Bhatwadekar et al. demonstrated that AGE-modification of vascular substrates reduces EPC attachment and spreading, abolishes EPCs chemotaxis, and reduces the ability of EPCs to repair wounded microvascular EC monolayers [70].

The mechanisms of endothelial dysfunction leading to DKD are summarized in Table 1. 

### 2.3. Therapies Targeting Endothelial Dysfunction in DKD 

Until recently, therapeutic interventions to prevent DKD onset and progression relied on four axes: glycemic control, cardiovascular risk reduction, blood pressure control, and RAS inhibition [3]. New anti-diabetic therapies have recently demonstrated a specific benefit on DKD, probably involving renal endothelial protection. Although anti-diabetic therapies do not specifically target endothelial cell injury, some of them may have beneficial effects on these cells.

#### 2.3.1. Glycemic Control and Diet Changes

Although genetic factors predispose diabetic patients to DKD, poor glycemic control is the main determinant of microalbuminuria and overt proteinuria, and glycemic control remains the first weapon against endothelial dysfunction and DKD in diabetic patients [3]. In type 1 diabetes, the DCCT group has demonstrated the benefit of intensive glycemic control for the prevention of DKD, with a reduction in the onset of microalbuminuria and overt proteinuria [71]. Intensive therapy also clearly reduces the incidence of cardiovascular events [72]. Although strict glycemic control in patients with type 2 diabetes is less beneficial than in type 1 diabetes [3], it has also been associated with a reduced risk of renal events (microalbuminuria and proteinuria) in the ADVANCE trial [73].

First-line therapy of type 2 diabetes is metformin, which is very effective in lowering blood glucose and could be nephroprotective for patients with DKD [74]. Beyond its anti-hyperglycemic properties, studies have shown beneficial effects of metformin therapy on endothelial cells. In endothelial cells, metformin decreases mitochondrial oxidative stress, increases NO bioavailability and reduces endothelial senescence and apoptosis [75]. These effects are probably mediated by reduced oxidative stress, as well as activation of the AMPK pathway leading to decreased mTOR signaling [75]. Clinical studies have provided evidence that metformin improves endothelium-dependent vasodilation in vivo [75]. 

Apart from glycemic control, diet changes could reduce the circulating levels of AGEs. Dietary proteins are an abundant exogenous source of AGEs [76], and in patients with DKD protein intake correlated with the urinary excretion of AGEs [77]. Reducing protein intake, which is a known reno-protective measure, could improve AGEs-related endothelial dysfunction.

The polyol pathway could be another interesting therapeutic target in DKD. Several FDA-approved drugs inhibit the polyol pathway: anti-oxidants, like α-lipoic acid, vitamin E, and vitamin C [78]. However, no randomized controlled trial has yet demonstrated a benefit of polyol pathway inhibition on DKD.

#### 2.3.2. Therapies Targeting Endothelial Injury as a Treatment for DKD?

Because of the crucial role of renal endothelial injury in DKD, some therapies targeting the mechanisms involved in this injury have been tested in clinical studies. These studies aimed to improve alteration of endothelial glycocalyx, to inhibit TGF-β, to decrease oxidative stress, and to increase NO bioavailability. However, most results have been disappointing to date. 

A therapeutic trial aiming to replenish glycocalyx through the ingestion of sulodexide (a mixture of glycosaminoglycans) in patients with type 2 diabetes failed to demonstrate a benefit compared to placebo on GFR decline or proteinuria [26,27]. In addition, whereas targeting TGF-β was promising in animals with DKD [41], TGFβ-1 inhibition by a monoclonal antibody failed to slow the progression of DKD in patients [42].

Clinical studies focused on decrease in oxidative stress or increase in NO bioavailability have also been performed. Folic acid, which improved NOS function and reduced progression of DKD in animal models, did not improve renal endothelial function or albuminuria in patients with DKD and microalbuminuria [79]. This result, in addition to the absence of beneficial effect of L-arginine or L-citrulline supplementation in diabetic mice [48], suggests that decreased NO bioavailability is more related to increased ROS rather than decreased NO. Therefore, a reduction of oxidative stress may improve NO bioavailability. Lowering the level of oxidative stress in DKD could have additional advantages, by reducing endothelial damage due to increased ROS production, and preventing the formation of the glucose by-products AGEs and sorbitol [46]. A recent meta-analysis evaluated the effect of antioxidants on the progression of DKD [80]. No clear benefit on GFR decline was documented, although a reduction in albuminuria was consistently noted [80]. Further studies are therefore needed to determine whether antioxidant therapies could be promising. 

#### 2.3.3. New Antidiabetic Therapies

Sodium glucose cotransporter 2 (SGLT2) inhibitors have been approved in the treatment of diabetes. They inhibit the uptake of glucose and sodium in the proximal tubule, leading to glycosuria and increased natriuresis, reducing hyperglycemia and favoring blood pressure control [3]. SGLT2 inhibitors also reverse hemodynamic changes observed in afferent and efferent arterioles, and may reduce glomerulosclerosis [81]. In two trials that demonstrated the benefit of SGLT2 inhibitors on cardiovascular risk reduction (EMPA-REG Outcome and CANVAS), positive effects were also observed on renal outcomes (40% reduction in the onset of albuminuria or composite renal outcome) [82]. Further trials, dedicated to renal outcomes, confirmed this benefit of SGLT2 inhibitors: the CREDENCE trial [83], conducted in patients with type 2 diabetes and CKD showed a 27% risk reduction in the composite renal outcome (end-stage renal disease, doubling of serum creatinine, or renal or cardiovascular death). 

Recent studies have shown that SGLT2 inhibitors are beneficial for the endothelium, notably through their anti-inflammatory and antioxidant effects [84,85]. SGLT2 inhibitors are associated with an improved systemic endothelial function assessed by FMD in diabetic patients [85]. This treatment partially ameliorates endothelium-dependent vasodilation in diabetic rats [86], probably via improved NO/cGMP signaling and reduced oxidative damage, rather than upregulation of eNOS expression [86]. Diabetic rodents treated with SGLT2 inhibitors display reduced vascular oxidative stress and inflammation [84,86,87], despite persistent hyperlipidemia and hyperinsulinemia [86]. Interestingly, the treatment of diabetic rats with SGLT2 inhibitors reduces AGE/RAGE signaling through an epigenetic regulation [86]. In cultured endothelial cells or in coronary artery segments, SGLT2 inhibitors improve endothelial viability [86], and attenuate hyperglycaemia-induced increase in endothelial senescence, oxidative stress, and inflammatory marker expression [88,89]. In vitro, hyperglycemia increases SGLT2 levels in endothelial cells, and SGLT-2 inhibitor reduces endothelial glucose uptake stimulated by hyperglycemia [88]. Therefore, treatment with SGLT2 inhibitors may reduce endothelial damage through the regulation of excessive glucose entry in endothelial cells and through the modulation of inflammation and oxidative stress in endothelial environment.

Dipeptidyl peptidase (DPP)-4 inhibitors, as well as glucagon-like peptide-1 receptor agonists (GLP-1RA), improve glycemic control through the increased blood levels of incretins, which stimulate insulin secretion and inhibit glucagon secretion. In the DELIGHT trial [90], the addition of saxagliptin, a DPP-4 inhibitor, to the SGLT2 inhibitor dapagliflozin increased the renal benefit (albuminuria) observed with dapagliflozin in patients with type 2 diabetes and moderate to severe CKD on stable doses of RAS inhibitor. DPP-4 inhibition is a promising tool to reduce kidney fibrosis [91].

In vitro, the DPP-4 inhibitor linagliptin suppresses endothelial expression of DPP-4 and integrin β1, thus reducing the assemblage of the TGF-β receptor and inhibiting EndMT [91]. DPP-4 inhibition also increased endothelial viability by promoting VEGF-R2 expression and inhibiting VEGF-R1 expression in endothelial cells [91]. 

GLP-1RA may also be promising to decrease diabetes-induced endothelial damage. GLP-1 RA mimic the effects of native GLP-1 binding on GLP-1 receptor, which is expressed at a low level in endothelial cells. GLP-1RA may prevent endothelial injury through the modulation of endothelial inflammation and oxidative stress [84]. GLP-1RA treatment of cultured endothelial cells reduces TNF-α-induced endothelial inflammation by a mechanism dependent on intracellular Ca2+ and activation of CAMKKβ and AMPK [92]. Activation of GPL-1 receptor has a protective effect on ROS-induced senescence of endothelial cells, through protein kinase A (PKA)-dependent activation of cAMP response element-binding (CREB) transcription factor and upregulation of antioxidant genes [93]. By activating GLP-1 receptor, GLP-1RA could reduce the detrimental effects of oxidative stress and inflammation in endothelial cells. 

Taken together, these studies show that DPP4 inhibition or GPL-1RA treatment may be promising therapies, beyond incretin-induced glycemic control, in the protection of renal endothelium in patients with diabetes.

## 3. Conclusions

In diabetes, high glucose and glucose by-products promote a pro-oxidant and pro-inflammatory environment that induces renal endothelial dysfunction. This dysfunction is a multifactorial process, notably encompassing increased permeability of glomerular endothelial cells, induction of endothelial apoptosis, glycocalyx breakdown, and impaired cross talk between endothelial cells and other renal cells like podocytes. Renal endothelial injury leads to albuminuria and renal fibrosis, with progressive CKD. In addition to glycemic control and RAS blockade, recent therapeutic strategies, which could also reduce endothelial damage, are effective in counteracting DKD initiation and/or progression.

## Figures and Tables

**Table 1:** Mechanisms of endothelial toxicity of high glucose and by products leading to diabetic kidney disease (DKD).

Mechanisms	Evidence	References
GEC damage	Increased GEC fenestration [20]. Increased GECs permeability [17] via PKC [11] and RAS activation [20]. Induction of GEC apoptosis [30].	[11,17,20,30]
Glycocalyx alterations	Reduced biosynthesis of glycosaminoglycans in GECs [22]. Increased synthesis of heparanase by GECs and podocytes [24].	[22,24]
Changes in VEGF pathways	Alteration of VEGF synthesis by podocytes [9,34,35]. Decreased VEGF expression in GEC [34].	[9,34,35]
Fibrosis	Stimulation of TGF-β secretion [4,38]. Induction of endothelial-mesenchymal transition (EndMT) [39,40].	[4,38,39,40]
Oxidative stress	Increased ROS production by NAD(P)H oxidase activation and eNOS uncoupling [45,49,51]. Increased mitochondrial ROS production by ECs [50] via endothelin-1 pathway [54]. Formation of AGEs further increasing NADPH-oxidase-dependent ROS synthesis [16,52].	[16,45,49,50,51,52,54].
Reduced NO bioavailability	Defect in eNOS expression [16], dimerization [17], and phosphorylation [17,47], leading to eNOS uncoupling. Decreased NO bioavailability due to increased oxidative stress and AGEs [4,16]. Depletion of BH_4_ co-factor in endothelial cells [4]. Elevation of ADMA [46].	[4,16,17,46,47]
Inflammation	Upregulation of endothelial adhesion molecule expression [4,57]. Increased expression of leukocyte counter-receptors [58]. Increased leukocyte adhesion to endothelial cells [58].	[4,57,58]
Decreased EPC repair capacities	Reduction of EPC angiogenic properties: impaired proliferation, migration, and incorporation in vascular structures [9,61,62,63,67]. Increased EPC senescence [63] and apoptosis [67]. Modification of vascular basement membrane leading to reduced EPC attachment and spreading [70]	[9,61,62,63,67,70]

## Abbreviations

List of abbreviations

ADMA	Asymmetric Dimethylarginine
AGEs	advanced glycation end products
BH_4_	tetrahydrobiopterin
COX-2	Cyclooxygenase-2
DKD	diabetic kidney disease
DPP-4	Dipeptidyl peptidase-4
EndMT	endothelial-mesenchymal transition
eNOS	endothelial *nitric oxide* synthase
EPC	endothelial progenitor cells
GECs	glomerular endothelial cells
GFR	glomerular filtration rate
GLP-1RA	glucagon-like peptide-1 receptor agonist
ICAM-1	Intercellular Adhesion Molecule 1
JNK	c-Jun NH2 terminal kinase
NADPH	nicotinamide *adenine* dinucleotide phosphate
NF-κB	nuclear factor-kappa B
NO	nitric oxide
PKC	protein kinase C
RAGE	receptor for advanced glycation endproducts
RAS	renin-angiotensin system
ROS	reactive oxygen species
SDF-1	stromal cell-derived factor 1
SGLT2	Sodium glucose cotransporter 2
TGF-β	transforming growth factor- β
VCAM-1	vascular cell adhesion molecule 1
VEGF	Vascular Endothelial Growth Factor
VEGFR2	Vascular Endothelial Growth Factor Receptor 2
